# Knowledge of Musculoskeletal Medicine in Junior Doctors in Australia: Is It Adequate?

**DOI:** 10.1007/s40670-022-01637-3

**Published:** 2022-09-23

**Authors:** Lahann Wijenayake, Sophie Conroy, Catherine McDougall, Paul Glasziou

**Affiliations:** 1 Orthopaedic Department, Queensland Childrens’ Hospital, Brisbane, QLD Australia; 2 The University of Queensland, Brisbane, Australia; 3 Princess Alexandra Hospital, Brisbane, QLD Australia; 4 Bond University, Gold Coast, QLD Australia

**Keywords:** Orthopaedic, Musculoskeletal, Education, Student

## Abstract

**Purpose:**

The incidence of musculoskeletal disease is increasing in Australia and around the world. However, medical student education does not necessarily reflect current and projected trends in musculoskeletal medicine. The aim of this study was to assess junior doctors’ competency in musculoskeletal medicine using the Freedman and Bernstein Basic Competency Examination in Musculoskeletal Medicine questionnaire.

**Methods:**

We conducted a cohort study of interns (first year post medical school) across four teaching hospitals in Australia. Interns were asked to take the Freedman and Bernstein examination during organised intern teaching sessions, and results were analysed using the original Freedman and Bernstein marking criteria and validated pass mark.

**Results:**

The mean score for the 92 interns was 13.9 out of 25 (55%) with scores ranging from 8 to 20.8 (29–83%). Only 8 of the 92 interns (8.7%) achieved a score of greater than 73%, the pre-specified pass mark.

**Conclusion:**

Our study identifies inadequacies in musculoskeletal medical knowledge in Australian interns. Review of undergraduate medical education may be required to reflect current and predicted trends in the prevalence of musculoskeletal disease and adequately prepare junior doctors.

## Introduction

The burden of musculoskeletal disease has risen dramatically over the past two decades. Musculoskeletal disorders are now considered the second most common cause of disability worldwide measured by years lived with disability (YLDs) [1]. An ageing population as well as increased rates of obesity and road traffic injuries suggest that the incidence of musculoskeletal diseases will continue to rise [2, 3].

As such, sound knowledge of musculoskeletal presentations is vital to achieving competency as a junior doctor. It is imperative that this area of clinical specialty is adequately addressed by medical school curricula and that junior doctors enter internship with a basic knowledge of how to approach these increasingly common disorders.

Australian statistics demonstrate 30% of Australians reporting musculoskeletal conditions in 2015 [4]. Overseas studies have found musculoskeletal injuries comprise approximately 15 to 30% of primary care visits, 20% of emergency room visits and 6% of general paediatric visits [5, 6].

In spite of this, limited time is often allocated to undergraduate teaching in musculoskeletal disorders. Currently, studies have estimated that the time allocated to musculoskeletal disease is less than 2% [5, 7, 8]. A number of previous studies have identified inadequacies in undergraduate education of musculoskeletal disease around the world [5, 7, 9, 10].

In 1998, Freedman and Bernstein developed a basic competency examination in musculoskeletal medicine administered to doctors in their first year post medical school (Table 1) [11]. The test was produced and later validated by the chairs of orthopaedic residency programmes in the USA who recommended a pass mark of 73%. The test is comprised of 25 short-answer questions, assessing a range of key topics including musculoskeletal anatomy, musculoskeletal emergencies and paediatric musculoskeletal conditions. This examination has been used internationally to assess the adequacy of musculoskeletal education in medical schools, with high failure rates seen in each instance [7, 11, 12].

**Table 1:** Freedman and Bernstein basic competency examination in musculoskeletal medicine with percentage of students who answered each question correctly

**Question**	Percentage of interns who answered correctly
**(1)** What common problem must all new newborns be examined for?	82%
**(2)** What is compartment syndrome?	55%
**(3)** Acute septic arthritis of the knee may be differentiated from inflammatory arthritis by which laboratory test?	43%
**(4)** A patient dislocates his knee in a car accident. What structure(s) is/are at risk for injury and therefore must be evaluated?	48%
**(5)** A patient punches his companion in the face and sustains a fracture of the 5^th^ metacarpal and a 3-mm break in the skin over the fracture. What is the correct treatment and why?	40%
(**6)** A patient comes to the office complaining of low back pain that wakes him up from sleep. What two diagnoses are you concerned about?	48%
**(7)** How is compartment syndrome treated?	83%
**(8)** A patient lands on his hand and is tender to palpation in the ‘snuff box’ (the space between the thumb extensor and abductor tendons). Initial radiographs do not show a fracture. What diagnosis must be considered?	82%
**(9)** A 25-year-old man is involved in a motor vehicle accident. His left limb is in a position of flexion at the knee and the hip, with internal rotation and adduction of the hip. What is the most likely diagnosis?	49%
**(10)** What nerve is compressed in carpal tunnel syndrome?	91%
**(11)** A patient had a disc herniation pressing on the 5^th^ lumbar nerve root. How is motor function of the 5^th^ lumbar nerve root tested?	29%
**(12)** How is motor function of the median nerve tested in the hand?	62%
**(13)** A 12-year-old boy severely twists his ankle. Radiographs show only soft-tissue swelling. He is tender at the distal aspect of the fibula. What are 2 possible diagnoses?	68%
**(14)** A patient presents with new-onset low back pain. Under what conditions are plain radiographs indicated? Please name 5 (example: history of trauma)	44%
**(15)** A patient has a displaced fracture near the fibula neck. What structure is at risk for injury?	53%
**(16)** A 20-year-old injured his knee while playing football. You see him on the same day, and he has a knee effusion. An aspiration shows frank blood. What are the 3 most common diagnoses?	59%
**(17)** What are the five most common sources of cancer metastases to bone?	95%
**(18)** Name two differences between rheumatoid arthritis and osteoarthritis	58%
**(19)** Which malignancy may be present in bone yet typically is not detected with a bone scan?	29%
**(20)** What is the function of the normal anterior cruciate ligament at the knee?	48%
**(21)** What is the difference between osteoporosis and osteomalacia?	32%
**(22)** In elderly patients, displaced fractures of the femoral neck are typically treated with joint replacement, whereas fractures near the trochanter are treated with plates and screws. Why?	58%
**(23)** What muscle(s) is/are involved in lateral epicondylitis (tennis elbow)?	27%
**(24)** Rupture of the biceps at the elbow results in weakness of both elbow flexion and which other movement?	44%
**(25)** What muscle(s) controls external rotation of the humerus with the arm at the side?	46%

The aim of this study was to assess the adequacy of musculoskeletal knowledge in Australian medical school graduates by applying the Freedman and Bernstein test to interns in their first post-graduate year.

## Methods and Materials

We enrolled interns at four teaching hospitals who were in their first post-graduate year in 2019. Ethics was obtained from the relevant ethics committee for all four sites. Verbal informed consent was obtained from all participants, and tests were de-identified so results remained anonymous. All participants took part voluntarily, and all interns who were approached agreed to take part in the study.

Inclusion criteria were interns, classified as Australian medical graduates in their first post-graduate year. Exclusion criteria included any overseas medical graduates and those who had previously undertaken the questionnaire.

Demographic data was also collected including which medical school the intern attended, estimated exposure to orthopaedics during medical school, self-expressed interest in specific specialties and self-assessed level of confidence in adequacy of own orthopaedic knowledge.

The Freedman and Bernstein examination involves 25 short-answer questions. There was no time limit to complete the test. The examination was marked according to the original validated scoring system by two independent markers (LW and SC). As recommended in the original study, the pass mark was set at 73%. Each of the 25 questions was worth one point, and the raw score out of 25 was multiplied by four to obtain a score between zero and 100 for each test.

Results were also categorised and assessed to identify particular areas of weakness or success. These sub-categories were anatomy, red-flag symptoms, and paediatrics. Pre-planned analysis was undertaken to assess the correlation between scores and clinical experience in orthopaedics. This included an assessment of scores by number of clinical days attended, number of hours of orthopaedic lectures received during medical school teaching, speciality of interest and self-assessed level of confidence.

## Results

### Demographics

A total of 92 interns were enrolled in the study representing graduates from 10 medical schools across Australia. The mean number of clinical days in orthopaedics attended during medical school, as reported by participants, was 18.8 days (range: 0–30 days). The mean number of lecture hours in orthopaedics attended during medical school, as reported by participants, was 7.4 h (range: 0–20 h). Ten of the 92 interns (10.8%) had completed an elective rotation in orthopaedics.

Only four interns (4.3%) nominated orthopaedics as their preferred future career choice; of note, 20 interns (22%) nominated emergency medicine and critical care, 15 interns (16%) nominated general practice and six interns (6.5%) nominated paediatrics. Regarding self-assessment of orthopaedic knowledge, five (5.5%) of the participants felt their level of orthopaedic knowledge was more than adequate for intern year, while 59 (64%) of the participants felt their knowledge adequate for intern year. Twenty-eight (31%) participants felt they had an inadequate level of knowledge in orthopaedics for an intern.

### Overall Score

**Fig. 1:**
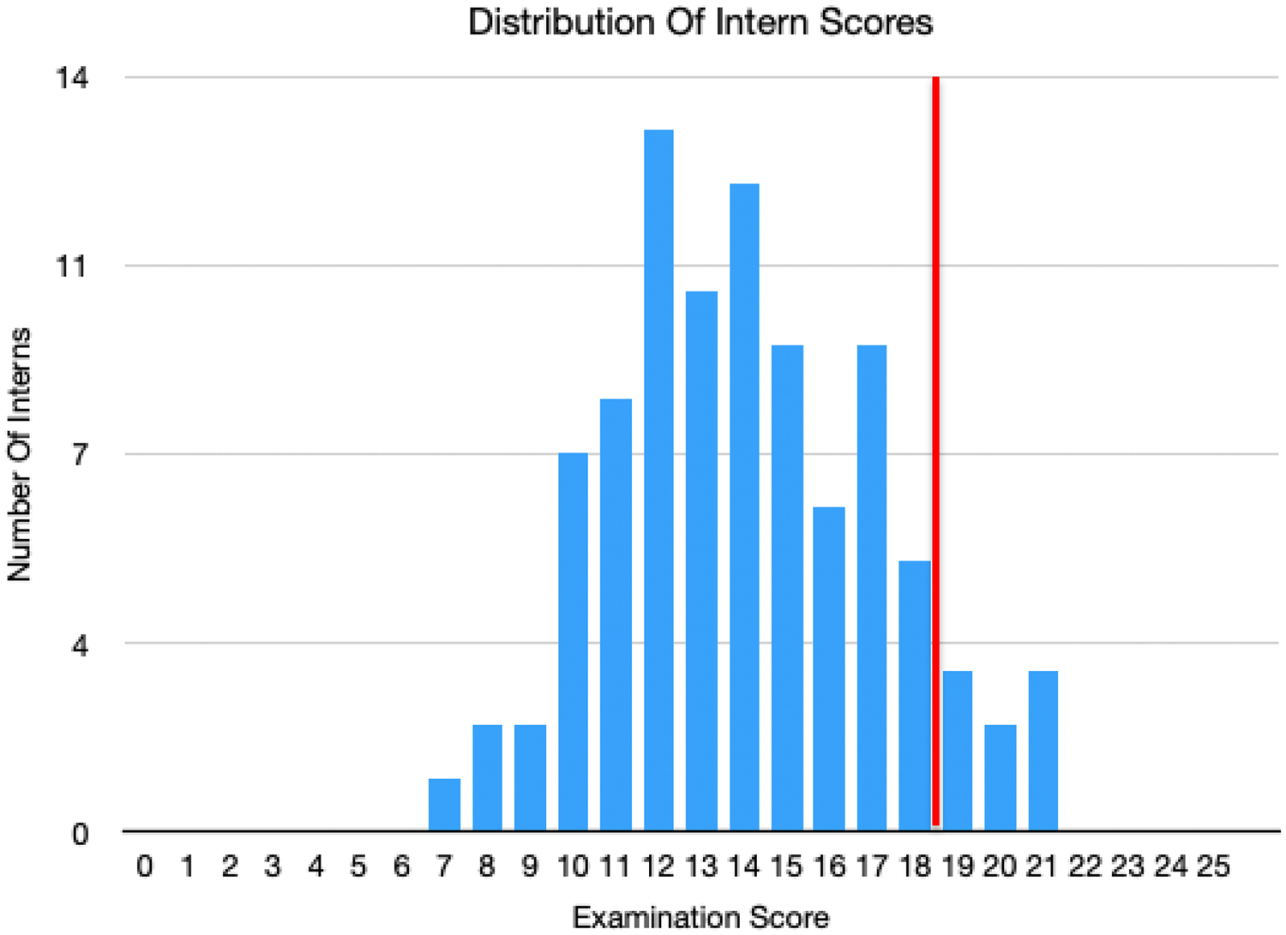
The mean score for the 92 interns was 13.9 out of 25 (56%) with overall scores ranging from 7 to 20.8 (29–83%). Red line highlights pass mark

The mean score for the 92 interns was 13.9 out of 25 (56%) with overall scores ranging from 8 to 20.8 (29–83%) (Fig. 1). Only 8 of the 92 interns (8.7%) achieved a score of greater than 73.1%, the pre-specified pass mark; therefore, 84 (92%) interns failed the examination. The highest-scoring question was question 17 (What are the five most common sources of cancer metastases to bone?), which was answered correctly by 95% of the interns. The lowest-scoring question was question 23 (What muscle(s) is/are involved in lateral epicondylitis/tennis elbow?), which was answered correctly by only 27% of the interns.

### Individual Component Scores

To assess specific areas of weakness or success, the original Freedman and Bernstein study categorised questions into anatomy-based questions (questions 8, 10, 11, 12, 15, 20, 22, 23, 24 and 25), red-flag questions (questions 2, 3, 4, 5, 6 and 7) and paediatric questions (questions 1 and 13). In this study, the anatomy-based questions had an average score of 54%, the red-flag questions had an average of 49% and the paediatric questions had a score of 75%.

### Correlation of Scores with Orthopaedic Experience

**Fig. 2:**
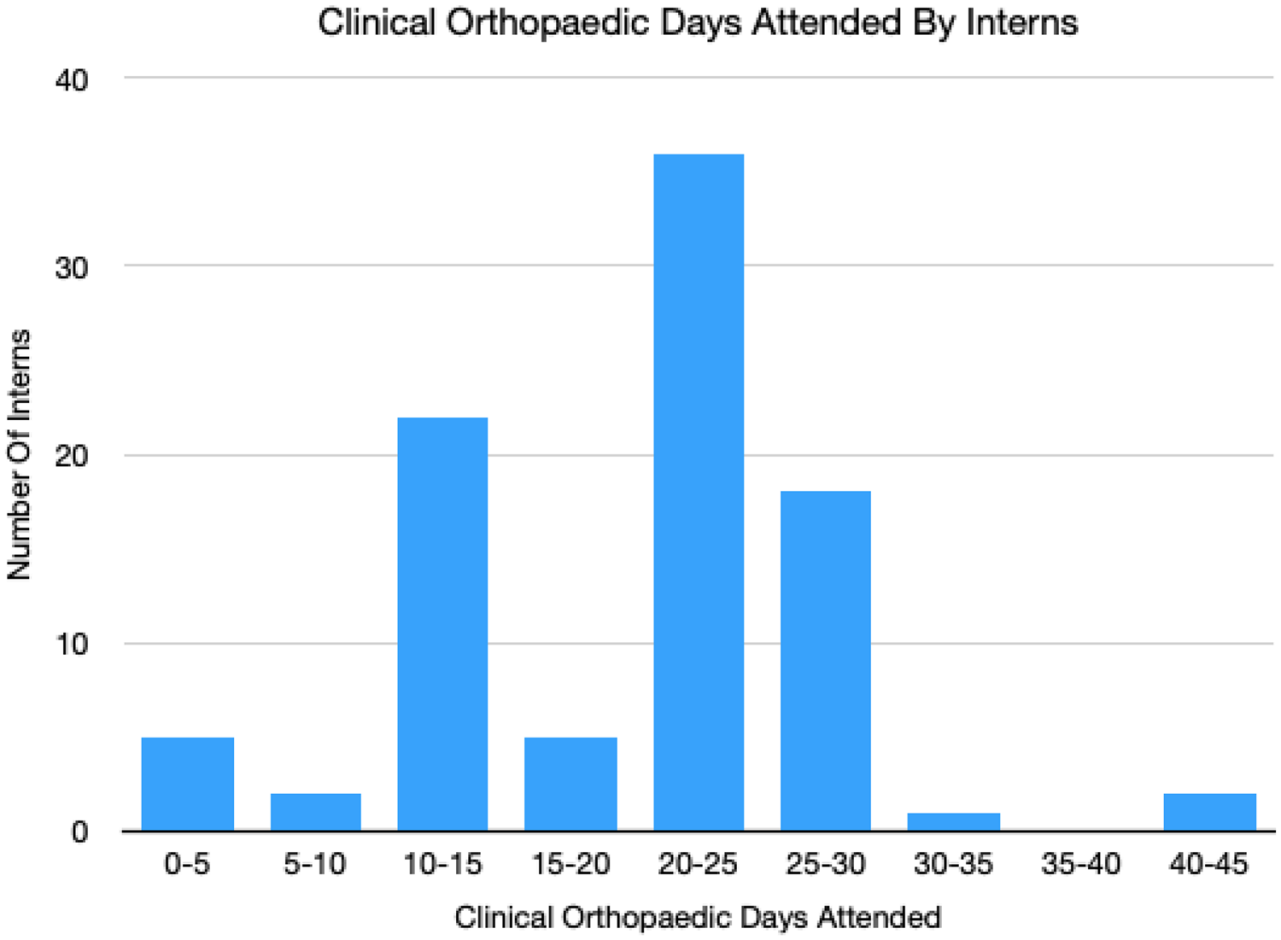
The average number of clinical orthopaedic days attended during the interns’ time at medical school was 18.8 days

The average number of clinical orthopaedic days attended during the interns’ time at medical school was 18.8 days, but we found 20 of the 92 participants (22%) had less than 10 days’ clinical experience in orthopaedics and 4 participants reported 0 days of orthopaedic clinical experience during medical school (Fig. 2). The average number of lecture hours specific to orthopaedics received during medical school training, as reported by the interns, was 7.4; however, 12 of the 92 participants (13%) reported no orthopaedic-specific lectures in their medical school training.

We found that the eight interns who passed the exam (i.e. achieved a mark of > 73%) on average attended 28.7 clinical days in orthopaedic rotations. This was 10 days more than the overall average of 18.4 days. The same eight interns who passed the exam reported they had attended an average of 11.3 lecture hours specific to orthopaedics throughout medical school. This amounted to 3.9 h more than the group average of 7.3 h of lecture time.

### Correlation of Scores with Speciality of Interest

Regarding prospective career choice, the interns who identified as considering a career in emergency medicine and critical care scored an average of 14.2 out of 25 (56%) on the exam. Those that nominated an interest in general practice had an average score of 13.5 out of 25 (54%). Interns who identified an interest in a career in paediatrics scored an average of 12.3 out of 25 (49%). Interns who were considering a career in a surgical stream scored 15.3 out of 25 (61%). All four interns who selected orthopaedics as a preferred career choice passed the test with an overall average score of 19.8 out of 25 (79%).

### Correlation of Scores with Self-Assessed Adequacy of Knowledge

Self-assessed adequacy of knowledge correlated with test scores. Participants who felt their orthopaedic knowledge was above the standard for intern level scored 19.9 out of 25 (79.6%), participants who thought they had an adequate level of knowledge scored 14.2 out of 25 (56.8%) and participants who assessed their knowledge to be below the adequate level for an intern scored 11.9 out of 25 (47.6%). This indicates that interns were aware of their strength or weakness in orthopaedic knowledge.

## Discussion

This study was the first Australian study to our knowledge to use the Freedman and Bernstein examination to assess junior doctor competency in musculoskeletal medicine. Using this validated tool helps identify areas for concern in intern knowledge and skills and can be used as a surrogate for adequacy of undergraduate medical education [10, 11].

The results of our study show reason for concern in Australian medical student orthopaedic education. Of great concern is the fact that only eight out of 92 (8.7%) interns achieved a score above the predetermined pass mark. The rising incidence of musculoskeletal disease is well established worldwide. The World Health Organization identified the years of 2000–2010 as the bone and joint decade (BJD) to reflect the increasing burden of chronic bone and joint diseases on health care systems worldwide. As such, sound knowledge of orthopaedic disease is vital to achieving competency as a junior doctor. It is imperative that this area of clinical specialty is adequately addressed by medical school curricula and that junior doctors enter internship with basic knowledge on approaching these increasingly common disorders.

Similar studies have been performed internationally using the Freedman and Bernstein examination. In the original study performed in Philadelphia in 1997, Freedman and Bernstein used their examination to assess 85 residents at the start of their first post-graduate year [11]. Seventy (82%) of the residents failed the basic competency examination in musculoskeletal medicine. In 2009, a British study assessed 112 interns at the end of their 2-year foundation programme. One hundred two of 112 (91%) interns failed the Freedman and Bernstein examination [7]. Dach et al. conducted a similar study in South Africa in 2010 and reported seven out of 79 (9%) achieved the validated exam pass mark [12].

As seen in the results, a number of interns had minimal clinical exposure or didactic teaching in the form of lectures, and some students even reported no clinical experience or orthopaedic-specific lectures. Unsurprisingly, clinical experience correlated with exam scores and those who scored best on the test reported a higher number of lecture hours and clinical days as compared with those who had lower scores.

Other studies have also shown increased competency, reflected by better exam scores, with increased clinical exposure and teaching. Day et al. demonstrated significantly better results on the Freedman and Bernstein questionnaire in students who completed an elective rotation [13]. This was reiterated by Dach et al. who showed a correlation between higher exam scores and medical student programmes with more time allocated to orthopaedic training [12]. The original Freedman and Bernstein study also demonstrated that devotion of more time in medical school rotations in orthopaedic surgery was associated with a better performance on the examination [11]. The development of a standardised curriculum as well as further engagement of clinicians as educators are some proposed methods to improve the knowledge of the students [13, 14].

In the original Freedman and Bernstein study, the breakdown of exam components into anatomy-based questions, red-flag questions and paediatric questions was to assess whether residents would perform better on questions of greater clinical importance, such as the red-flag questions. The red-flag questions include topics such as compartment syndrome, vascular injury and septic arthritis. Concerningly, this was the area in our study with the poorest results. For example, only 55% of the interns knew the definition of compartment syndrome and only 49% knew that the popliteal artery could be injured in a knee dislocation. Both of these injuries are limb threatening.

Unsurprisingly, our results showed that interns who had an interest in orthopaedics and elected this as their preferred career passed the assessment. Furthermore, those who had an interest in a surgical stream did above average. However, those intending to pursue emergency medicine and general practice scored below average, both areas with large components of musculoskeletal presentations [5]. Self-assessment of knowledge adequacy correlated with the results, and 30% of participants identified an inadequate level of orthopaedic knowledge; these participants on average scored below the pass mark and below the average for all students. More concerning, however, was the 59% of participants who assessed the level of orthopaedic knowledge as being adequate; on average, these interns scored 56%, 17% below the pre-specified pass mark.

### Limitations

There are limitations to our study. Firstly, the Freedman and Bernstein examination itself has some limitations. The original authors recognised that the validity is limited by the open response formatting, the wording of the questions and the accepted answers [11]. Furthermore, our sample was taken from four hospitals and may not be truly representative of the Australian medical school system. However, students from ten of the 18 medical schools around Australia were represented in the study. Additionally, our study relied on participants to recall and accurately report their clinical experience in terms of clinical days and lecture hours, and it is difficult to assess how accurate these estimations are.

## Conclusion

The findings of this study highlight some inadequacy in knowledge of musculoskeletal disorders in junior doctors in Australia. The ability of junior doctors to be able to diagnose and manage these conditions is increasingly important as the prevalence of musculoskeletal disorders increases. Our study supports growing evidence to suggest there needs to be improvements in undergraduate medical education to reflect current trends in musculoskeletal disease to adequately prepare junior doctors. Increasing clinical exposure and teaching time specifically devoted to orthopaedics is likely to improve knowledge and competency in orthopaedics and musculoskeletal medicine among junior doctors. This in turn will improve health-related patient outcomes by improving the quality of health care provided by junior doctors.

## Data Availability

Not applicable.
